# Post-translational modification enzymes as key regulators of ciliary protein trafficking

**DOI:** 10.1093/jb/mvab024

**Published:** 2021-03-03

**Authors:** Taro Chaya, Takahisa Furukawa

**Affiliations:** Laboratory for Molecular and Developmental Biology, Institute for Protein Research, Osaka University, Osaka 565-0871, Japan

**Keywords:** ICK/CILK1, kinase, MAK, retina, ubiquitin

## Abstract

Primary cilia are evolutionarily conserved microtubule-based organelles that protrude from the surface of almost all cell types and decode a variety of extracellular stimuli. Ciliary dysfunction causes human diseases named ciliopathies, which span a wide range of symptoms, such as developmental and sensory abnormalities. The assembly, disassembly, maintenance and function of cilia rely on protein transport systems including intraflagellar transport (IFT) and lipidated protein intraflagellar targeting (LIFT). IFT is coordinated by three multisubunit protein complexes with molecular motors along the ciliary axoneme, while LIFT is mediated by specific chaperones that directly recognize lipid chains. Recently, it has become clear that several post-translational modification enzymes play crucial roles in the regulation of IFT and LIFT. Here, we review our current understanding of the roles of these post-translational modification enzymes in the regulation of ciliary protein trafficking as well as their regulatory mechanisms, physiological significance and involvement in human diseases.

## Graphical Abstract

**Fig. 1.**
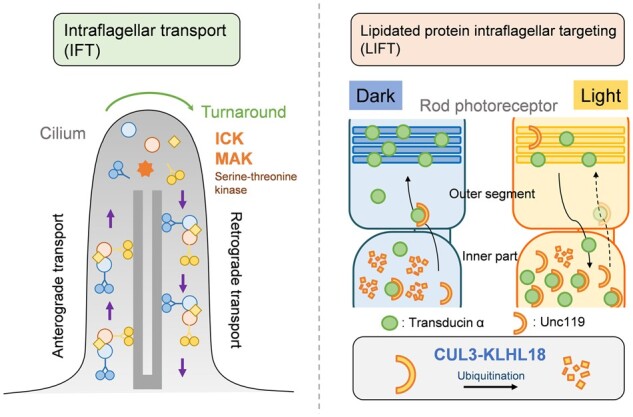
**The serine-threonine kinases MAK and ICK in the regulation of IFT**. (**A**) Phylogenetic distribution of protein kinases in humans. MAK and ICK are classified into the CMGC kinase family. TK, Tyrosine kinase; TKL, Tyrosine kinase-like; STE, Homologs of yeast Sterile 7, Sterile 11, Sterile 20 kinases; CK1, Casein kinase 1; AGC, Containing PKA, PKG, PKC families; CAMK, Calcium/calmodulin-dependent protein kinase; CMGC, Containing CDK, MAPK, GSK3, CLK families. Numbers of kinases classified into each family are indicated (110). (**B**) Proposed model of the regulation of IFT turnaround at the ciliary tip by MAK and ICK.

The primary cilium is a hair-like microtubule-based structure that protrudes from the surface of nearly every cell and performs a wide range of sensory functions across species (1, 2). The ciliary membrane and interior are distinguished from the plasma membrane and the cytoplasm, respectively, by the presence of the transition zone and the transition fibres (3). A variety of signalling receptors localize to primary cilia and receive extracellular stimuli including light, odorants and Hedgehog morphogens. For example, retinal photoreceptor cells develop the outer segment, a light-sensory structure containing components of the phototransduction cascade including photopigments, which are formed initially from the primary cilia in photoreceptor precursors (4). Therefore, cilia function as a signalling centre for multiple transduction pathways. Ciliary dysfunction leads to human diseases termed ‘ciliopathies’, which are characterized by a broad spectrum of symptoms, including polydactyly, cranio-facial abnormalities, brain malformation, obesity, polycystic kidney, anosmia, hearing loss and retinal degeneration (5–7).

## Intraflagellar Transport

The biogenesis and maintenance of cilia depend on intraflagellar transport (IFT), the bidirectional protein trafficking system coordinated by three large protein complexes, IFT-B, IFT-A and the BBSome, with molecular motors along the axonemal microtubules of cilia. They assemble into linear arrays, known as IFT trains, which mediate ciliary protein transport along the axoneme in both anterograde and retrograde directions (8). In addition, the three IFT complexes function in ciliary import and export of proteins (9, 10). IFT-B, a 16-subunit complex, mediates anterograde transport from the base to the tip driven by the kinesin-2 motor, whereas IFT-A, a 6-subunit complex, mediates retrograde transport from the tip back to the base driven by the cytoplasmic dynein-2 motor (9, 11). The BBSome, an 8-subunit complex, functions as a coat adaptor for IFT-B and mediates the exit of membrane proteins including GPCRs from cilia (9, 12, 13). In addition, it also assists the assembly of IFT trains at the ciliary base (14). At the ciliary tip, IFT trains are disassembled and then reassembled for turnaround and retrograde transport (15). Mutations in some genes encoding components of IFT trains are known to be associated with ciliopathies including Bardet-Biedl syndrome (BBS) and Joubert syndrome (16).

## Serine–Threonine Kinases MAK and ICK in the Regulation of Ciliary Protein Trafficking

Various serine**–**threonine kinases play crucial roles in many aspects of cilia assembly, disassembly, maintenance and function. Two serine-threonine kinases from a branch of CDK/MAPK/GSK3/CLK (CMGC) kinases, male germ cell-associated kinase (MAK) and intestinal cell kinase (ICK), also known as ciliogenesis-associated kinase 1 (CILK1), are proposed to serve as regulators of IFT turnaround at the ciliary tip and ciliary length in mammalian cells (Fig. 1). Since IFT plays a key role in ciliary length control, regulation of IFT is thought to be important for the ciliary length regulation (17). As the names imply, *Mak* and *Ick* were originally identified in male germ cells and intestinal crypt cells, respectively (18, 19). MAK and ICK are evolutionarily conserved mitogen-activating protein kinase-like kinases that exhibit high homology, especially in their catalytic domains (19–21). *Mak* is predominantly expressed in the retina and testis whereas *Ick* is ubiquitously expressed among tissues (22). In contrast to the distinct tissue distribution patterns, these protein kinases show a similar subcellular localization. MAK localizes to the distal region of ciliary axonemes in retinal photoreceptor cells (23). ICK localizes mainly to the ciliary tip, which is mediated through the anterograde trafficking by IFT-B (24, 25). *Caenorhabditis elegans* DYF-5, an orthologue of MAK and ICK, is an IFT cargo molecule transported to the distal segments of sensory cilia (26). In addition, ICK is enriched in ciliary vesicles released from the tip of cilia (27). *Mak*-deficient mice exhibit elongated photoreceptor ciliary axonemes with the accumulation of IFT88, an IFT-B component, at the distal portion (23). Loss-of-function of ICK causes shortened or elongated cilia, impaired Hedgehog signalling and accumulation of IFT-B, IFT-A and BBSome components at ciliary tips, while ICK overexpression induces accumulation of IFT-B, but not IFT-A and BBSome components at the tip of cilia, suggesting roles of ICK in disassembly of IFT trains in the turnaround process (24, 25, 28–32). *Chlamydomonas reinhardtii* (*Chlamydomonas*) LF4, *Tetrahymena* LF4A, *Leishmania mexicana* LmxMPK9, and *C. elegans* DYF-5, orthologues of MAK and ICK, are also involved in the regulation of IFT as well as cilia/flagella length and formation (26, 33–37).

**Fig. 1.**
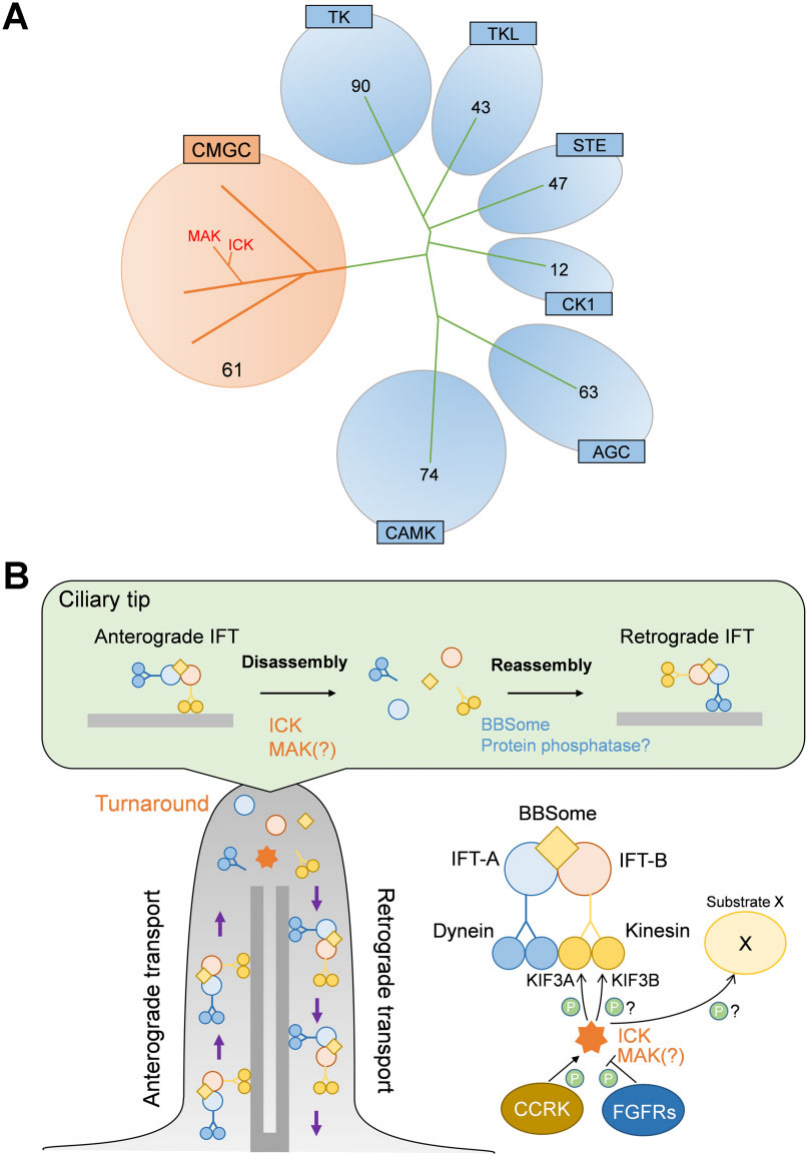
**The serine-threonine kinases MAK and ICK in the regulation of IFT**. (**A**) Phylogenetic distribution of protein kinases in humans. MAK and ICK are classified into the CMGC kinase family. TK, Tyrosine kinase; TKL, Tyrosine kinase-like; STE, Homologs of yeast Sterile 7, Sterile 11, Sterile 20 kinases; CK1, Casein kinase 1; AGC, Containing PKA, PKG, PKC families; CAMK, Calcium/calmodulin-dependent protein kinase; CMGC, Containing CDK, MAPK, GSK3, CLK families. Numbers of kinases classified into each family are indicated (110). (**B**) Proposed model of the regulation of IFT turnaround at the ciliary tip by MAK and ICK.

ICK phosphorylates the C-terminal portion of KIF3A, a subunit of kinesin-2, including Thr674 (24, 38). KIF3A Thr674 is positioned in a consensus amino acid sequence for MAK and ICK phosphorylation that is evolutionarily conserved (39). Localization of KIF3A phosphorylated at Thr674 to ciliary tips is observed in mouse embryonic fibroblasts, which is attenuated by *Ick* deficiency. Inhibition of phosphorylation on serine/threonine residues including Thr674 at the KIF3A C-terminal portion perturbs cilia formation in cultured cells and zebrafish (24). Mouse embryonic fibroblasts carrying a Thr-to-Ala mutation at residue 674 on KIF3A (KIF3A T674A) show slightly elongated cilia without affecting ciliary localization of IFT88 (40). These observations suggest that ICK phosphorylates other substrate protein(s) in addition to the KIF3A C-terminal portion including Thr674 to regulate IFT and ciliary length. In *Chlamydomonas*, phosphorylation of the kinesin-2 motor subunit FLA8, an orthologue of KIF3B, at Ser663 is required for IFT turnaround at the flagellar tip (41). Intriguingly, FLA8 Ser663 is located in a consensus amino acid sequence for phosphorylation by MAK and ICK that is evolutionarily conserved among species, suggesting that IFT turnaround at the ciliary tip is mediated by phosphorylation of KIF3B in addition to KIF3A by MAK and ICK in mammals. *C. elegans* hypomorphic mutants of *bbs-1*, a gene encoding a BBSome component, show accumulation of IFT-B but not IFT-A components at the ciliary tip, as similarly observed in ICK overexpressing cells (14). Given that the BBSome assembles IFT trains at the ciliary base, disassembly and reassembly of IFT trains at the ciliary tip may be mediated by ICK, as well as probably MAK, and the BBSome, respectively.

## MAK and ICK in Development, Physiology and Disease

*Mak*-deficient mice are viable and fertile without obvious developmental abnormalities, but exhibit progressive retinal photoreceptor degeneration (23). Interestingly, mutations in human *MAK* gene were discovered in patients with autosomal recessive retinitis pigmentosa (RP), a retinal degenerative disease (42, 43). In contrast, *Ick*-deficient mice exhibit neonatal lethality accompanied with developmental abnormalities observed in multiple organ systems including the bone, lung, kidney, brain, retina, and inner ear (44). In humans, two homozygous loss-of-function mutations in *ICK* gene, R272Q and G120C, are associated with endocrine-cerebro-osteodysplasia (ECO) syndrome, an autosomal recessive ciliopathy characterized by neonatal lethality with multiple defects involving the endocrine, cerebral, and skeletal systems (31, 45). Another homozygous loss-of-function mutation in human *ICK* gene, E80K, is associated with short rib-polydactyly syndrome (SRPS), an autosomal recessive ciliopathy showing perinatal lethality with short ribs, shortened and hypoplastic long bones, polydactyly and multiorgan system abnormalities (30). In addition, heterozygous variants in human *ICK* gene are linked to juvenile myoclonic epilepsy (46). Of them, four strongly linked variants, K220E, K305T, A615T and R632X, affect ICK functions in cilia formation and impair mitosis, cell-cycle exit and radial neuroblast migration while promoting apoptosis in the cerebral cortex (46, 47). In contrast to *Ick*-deficient mice, homozygous KIF3A T674A knock-in mice exhibit mildly reduced alveolar airspace in the lung, but are viable without gross abnormalities in the bone and brain, suggesting that other substrate(s) in addition to KIF3A Thr674 are phosphorylated by ICK *in vivo*.

## Regulatory Mechanisms of MAK and ICK Activities

MAK and ICK are activated by phosphorylation at the TDY motif in their kinase domain by cell cycle-related kinase (CCRK) *in vitro* (39). ICK phosphorylation by CCRK negatively regulates ciliogenesis in cultured cells (48). Similar to loss of *Mak* or *Ick*, *Ccrk* deficiency causes dysregulation of cilia length and accumulation of IFT88 at ciliary tips (49). *Ccrk*-deficient mice show multiple developmental abnormalities associated with dysregulation of Hedgehog signaling, including polydactyly, neural tube patterning defects and malformation of the lung and eye (49–51). CCRK orthologues, *Chlamydomonas* LF2 and *C. elegans* DYF-18, also participates in the IFT regulation as well as cilia/flagella length and formation control (26, 36, 52, 53). LF4 phosphorylation at the TDY motif is diminished in the *Chlamydomonas lf-2* mutant, suggesting that the LF2-LF4 signaling axis is evolutionarily conserved among species (54). In contrast to CCRK, fibroblast growth factor (FGF) signalling is thought to negatively regulate the ICK activity. Inactivation of Fgf receptor 1 (Fgfr1) or its ligands results in shortened cilia in zebrafish and *Xenopus* (55). On the other hand, FGFR3 activation shortened cilia and perturbed the localization of IFT20, an IFT-B component, to cilia in mammals (56, 57). Biochemical experiments show that FGFRs interact with, phosphorylate and inactivate ICK. In cultured cells, FGF treatment modulates cilia length through ICK (58).

## Lipidated Protein Intraflagellar Targeting in Retinal Photoreceptor Cells

In parallel to IFT, lipidated protein intraflagellar targeting (LIFT) plays crucial roles in establishing a dynamic ciliary signaling compartment (59). Transport of some lipidated proteins into cilia relies on specific chaperones uncoordinated 119 (UNC119) and phosphodiesterase 6δ (PDE6δ), which directly recognize lipid chains in the cytoplasmic region and unload their cargoes inside cilia upon interacting with the small ARF-like GTPase protein ARL3 bound to GTP (60–63). ARL3 is converted from its inactive GDP-bound state to the active GTP-bound state inside cilia by ARL13B, a guanine nucleotide exchange factor (GEF) constitutively localized to cilia (64). In contrast, retinitis pigmentosa 2 (RP2), a GTPase-activating protein (GAP) localized at the base of cilia, is predicted to keep ARL3 in its GDP-bound state outside cilia (65, 66).

Molecular mechanisms and physiological roles of LIFT have been well studied in retinal rod and cone photoreceptor cells. Rod photoreceptor cells are sensitive to light and are responsible for low light vision, while cone photoreceptor cells operate at a brighter range of light intensities and are responsible for high-resolution daylight and colour vision. Subcellular localization of rod transducin, a heterotrimeric G protein that is a mediator of the phototransduction cascade, changes responding to ambient light (67–71). The inner part and outer segment of the photoreceptor cell are connected by a connecting cilium. Rod transducin is transported to the outer segment from the inner part through the connecting cilium, and then concentrated in the outer segment under dark-adapted conditions. After light reception, rod transducin translocates from the outer segment to the inner part through the connecting cilium. This light- and dark-dependent translocation of transducin modulates photosensitivity in rod photoreceptors, thereby contributing to light and dark adaptation. Localization of the α-subunit of rod transducin (rTα) to the outer segment is required for normal light sensitivity of rod photoreceptors (72). In contrast, light-dependent rTα translocation from the outer segment to the inner part protects the retina from the light-induced damage (73–76). UNC119 interacts with the acylated N termini of rTα and suppresses rhodopsin-mediated transducin activation (63, 77). The dark-triggered transport of rTα to the outer segment is inhibited in the *Unc119*-deficient mouse retina (63). Deletion of *unc-119* in *C. elegans* also blocks G protein trafficking to cilia, suggesting an evolutionarily conserved role of UNC119 (63). *Unc119*-deficient mice exhibit a slowly progressive retinal degeneration (78). A heterozygous stop codon (K57X) was found in the human *UNC119* gene of an individual with late-onset dominant cone dystrophy (79). Transgenic mice carrying the identical mutation show mitochondrial ANT-1-mediated retinal degeneration (79, 80). On the other hand, PDE6δ, encoded by *Pde6d*, is a prenyl-binding protein that promotes translocation of the βγ-subunit of rod transducin (rTβγ) from the inner part to the outer segment. In the *Pde6d^−/−^* mouse retina, rTγ, known to be farnesylated, mislocalizes to the inner part (81, 82). In addition to rTγ, several components of the phototransduction cascade are also prenylated in rod and cone photoreceptors. PDE6α and PDE6β, rod cGMP phosphodiesterase catalytic subunits, PDE6α’, a cone cGMP phosphodiesterase catalytic subunit and rhodopsin kinase (GRK1) are farnesylated or geranylgeranylated (83–85). Reduced localization of GRK1 and PDE6α’ to the outer segment as well as mislocalization of rod PDE6 subunits to the inner part are observed in *Pde6d^−/−^* rod and cone photoreceptors (82). As a consequence, loss of *Pde6d* results in altered electrophysiological properties of photoreceptor cells and a slowly progressing retinal degeneration (82). Consistent with *Unc119* and *Pde6d* deficiency, *Arl3* conditional knockout mice show trafficking defects of lipidated proteins including rTα, rTγ, rod PDE6 and GRK1 to outer segments in rod photoreceptors and subsequent retinal degeneration (86). A mutation in the human *ARL3* gene is linked to autosomal dominant RP (87). Deletion of *Arl13b* in the mouse retina leads to mislocalization of rTα and rod PDE6 subunits but faster retinal degeneration than *Arl3* deficiency does, suggesting additional functions of ARL13B other than a GEF for ARL3 in rod photoreceptor cells (88, 89). In humans, mutations in the *ARL13B* gene are associated with Joubert syndrome, an autosomal recessive ciliopathy characterized by multiple symptoms including retinal degeneration (90, 91). *Rp2*-deficient mice exhibit defects in trafficking of GRK1 as well as rod and cone PDE6 to the outer segment, and subsequent slowly progressing retinal degeneration (92). Mutations in the human *RP2* gene are associated with X-linked RP, macular atrophy and cone-rod dystrophy (93–95).

## Regulation of Transducin Translocation During Light-Dark Adaptation by the CUL3-KLHL18 Ubiquitin Ligase

**Fig. 2.**
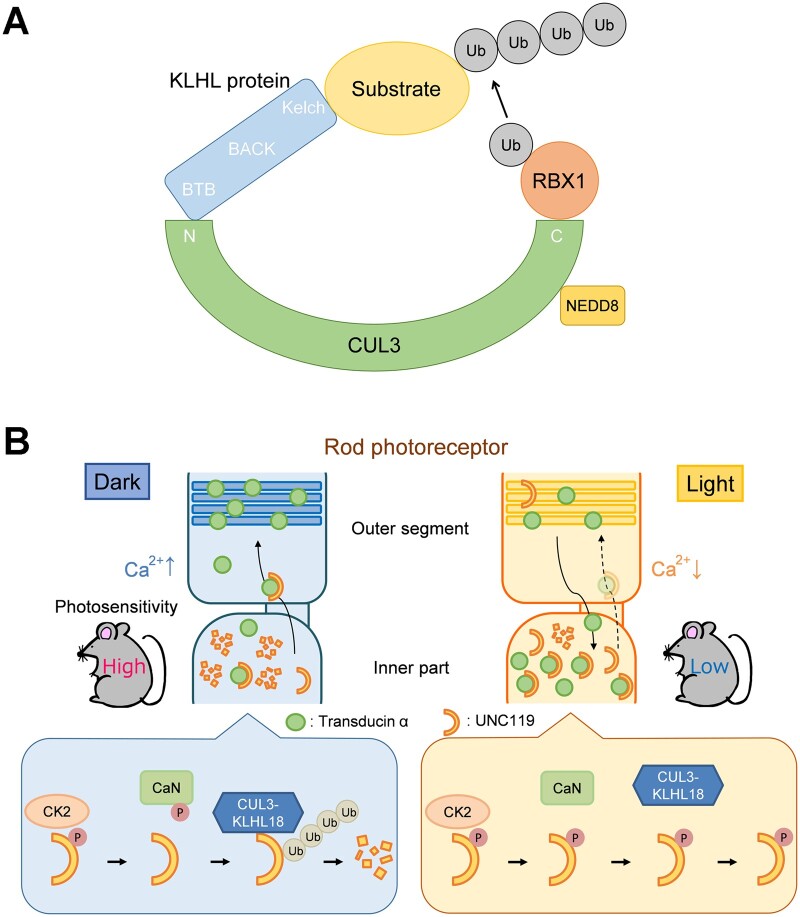
**The CUL3-KLHL18 ubiquitin ligase in the regulation of LIFT**. (**A**) Schematic diagram of CUL3-KLHL ubiquitin ligases. (**B**) Proposed model of the α-subunit of rod transducin (rTα) translocation during light and dark adaptation in rod photoreceptor cells. In rod photoreceptors, intracellular Ca^2+^ concentration under dark conditions is higher than that under light conditions. In darkness, CK2-phosphorylated UNC119 is dephosphorylated by calcineurin, a Ca^2+^-dependent phosphatase. CUL3-KLHL18 efficiently ubiquitinates and degrades the dephosphorylated UNC119, thereby facilitating rTα transport from the inner part to the outer segment. In the light, calcineurin-mediated dephosphorylation of CK2-phosphorylated UNC119 is suppressed due to low Ca^2+^ concentration. Inefficient ubiquitination and degradation of the phosphorylated UNC119 by CUL3-KLHL18 results in increased amounts of UNC119 and subsequent inhibition of rTα translocation to the outer segment. CaN, calcineurin.

The ubiquitin proteasome system is one of the fundamental regulatory tools used by eukaryotic cells. Cullin-RING (really interesting new gene) ubiquitin ligases (CRLs) form one of the largest groups of ubiquitin E3 ligases that regulate diverse cellular pathways (96). Cullin-3 (CUL3) bridges the interaction between the RING protein RBX1 and substrate adaptors which deliver targets for ubiquitination and proteasomal degradation (97). Covalent attachment of the ubiquitin-like protein NEDD8 to Cullin family proteins is required for the activation of the Cullin-based ubiquitin E3 ligases (98). The N-terminus of CUL3 interacts with the Broad Complex, Tramtrack, Bric-a-Brac (BTB) domain of substrate adaptors including the Kelch-like (KLHL) family proteins, which contain one BTB domain, one BTB and C-terminal kelch (BACK) domain, and five to six Kelch repeats (Fig. 2A) (99). The HUGO Gene Nomenclature Committee (HGNC) presently defines 42 KLHL genes.

Recently, *Kelch-like 18* (*Klhl18*), one of the *Klhl* genes, was found to be predominantly expressed in retinal photoreceptor cells. *Klhl18*-deficient mice exhibit decreased light responses in rod photoreceptors and rTα mislocalization from the outer segment to the inner part. Loss of *Klhl18* or treatment of MLN4924, a small molecule inhibitor of the NEDD8-activating enzyme (NAE) (100), suppresses light-induced retinal degeneration. The CUL3-KLHL18 ubiquitin ligase ubiquitinates and degrades UNC119 in rod photoreceptor cells, preferentially under dark conditions. UNC119 overexpression phenocopies the rTα mislocalization observed in the *Klhl18^−/−^* mouse retina. These observations suggest that CUL3-KLHL18 modulates rTα translocation during light and dark adaptation through UNC119 ubiquitination and degradation. Furthermore, regulatory mechanisms underlying the CUL3-KLHL18-UNC119 axis were investigated. The phosphorylation level of UNC119 is elevated under light conditions compared with that under dark conditions. UNC119 is phosphorylated by casein kinase 2 (CK2), which is a serine/threonine kinase expressed in retinal photoreceptor cells (101). In contrast, UNC119 is dephosphorylated by Calcineurin, a Ca^2+^- and calmodulin-dependent serine/threonine protein phosphatase (102, 103). UNC119 degradation by CUL3-KLHL18 is suppressed and facilitated by UNC119 phosphorylation and dephosphorylation, respectively. Inhibition of CK2 decreases the amount of UNC119 in the retina, while inhibition of Calcineurin increases the retinal expression level of UNC119, causes rTα mislocalization to the inner part of photoreceptors, and protects the retina from light-induced damage. Collectively, these results suggest that CUL3-KLHL18 promotes UNC119 ubiquitination and degradation depending on phosphorylation, thereby regulating light- and dark-dependent rTα translocation in the retina (Fig. 2B) (104). Given that light exposure is a suspected risk factor for the progression of age-related macular degeneration and RP (105–108), inhibition of this signaling pathway may be a potential therapeutic target.

## Concluding Remarks

It has recently become clear that several post-translational modification enzymes play key roles in the regulation of ciliary protein trafficking. Cumulative evidence of the functional mechanisms of MAK and ICK has unravelled many aspects of the physiological significance of the coordinated IFT turnaround at the ciliary tip and its involvement in human ciliopathies. Identification and functional characterization of the CUL3-KLHL18 ubiquitin ligase in retinal photoreceptor cells put forward our knowledge of a series of regulatory mechanisms from photoreception to light-dark adaptation. However, these findings raise new questions. For example, assuming that IFT trains are disassembled by MAK and ICK at the ciliary tip, how are they reassembled for retrograde IFT? In parallel to the BBSome as mentioned above, serine-threonine phosphatase(s) may also contribute to reassembly of IFT trains at the ciliary tip through dephosphorylating KIF3A and other substrate(s). In addition, although rTβγ also shows light- and dark-dependent translocation between the outer segment and the inner part of retinal photoreceptor cells, which modulates rod light responses (109), rTβγ translocation is independent of CUL3-KLHL18. PDE6δ ubiquitination and degradation by an unknown E3 ubiquitin ligase may underpin rTβγ translocation during light and dark adaptation. Further analyses are needed to unveil comprehensive regulatory mechanisms underlying ciliary protein trafficking, whose understanding would contribute to develop therapeutic strategies to treat human diseases.

## Funding

This work was supported by Grant-in-Aid for Scientific Research (18H02593, 20K07326) from the Japan Society for the Promotion of Science, The Cell Science Research Foundation, Suzuken Memorial Foundation, The Uehara Memorial Foundation, and The Takeda Science Foundation. 

## Conflict of interest

None declared. 

## Abbreviations

BBS: Bardet-Biedl syndrome
CK2: casein kinase 2
FGF: fibroblast growth factor
IFT: intraflagellar transport
LIFT: lipidated protein intraflagellar targeting.
